# A Systematic Review of Telemedicine-Driven Pulmonary Rehabilitation after the Acute Phase of COVID-19

**DOI:** 10.3390/jcm12144854

**Published:** 2023-07-24

**Authors:** Camelia Corina Pescaru, Alexandru Florian Crisan, Monica Marc, Ana Adriana Trusculescu, Adelina Maritescu, Andrei Pescaru, Anastasiia Sumenkova, Felix Bratosin, Cristian Oancea, Emanuela Vastag

**Affiliations:** 1 Center for Research and Innovation in Personalized Medicine of Respiratory Diseases (CRIPMRD), “Victor Babes” University of Medicine and Pharmacy Timisoara, Eftimie Murgu Square 2, 300041 Timisoara, Romania; pescaru.camelia@umft.ro (C.C.P.); marc.monica@umft.ro (M.M.); ana.trusculescu@umft.ro (A.A.T.); oancea@umft.ro (C.O.); tudorache_emanuela@yahoo.com (E.V.); 2 Pulmonology Clinic, Clinical Hospital of Infectious Diseases and Pulmonology, “Victor Babes”, Gheorghe Adam Street 13, 300310 Timisoara, Romania; adelina.maritescu@umft.ro; 3 Research Center for the Assessment of Human Motion, Functionality and Disability (CEMFD), “Victor Babes” University of Medicine and Pharmacy Timisoara, Eftimie Murgu Square 2, 300041 Timisoara, Romania; 4 Doctoral School, “Victor Babes” University of Medicine and Pharmacy Timisoara, Eftimie Murgu Square 2, 300041 Timisoara, Romania; felix.bratosin@umft.ro; 5 Faculty of General Medicine, “Victor Babes” University of Medicine and Pharmacy Timisoara, Eftimie Murgu Square 2, 300041 Timisoara, Romania; andrei.pescaru@student.umft.ro; 6 Penza State University, Faculty of Medicine, Ulitsa Krasnaya 40, 440026 Penza, Russia; md16as@gmail.com; 7 Department XIII, Discipline of Infectious Diseases, University of Medicine and Pharmacy “Victor Babes” Timisoara, Eftimie Murgu Square 2, 300041 Timisoara, Romania

**Keywords:** coronavirus, COVID-19, telerehabilitation, virtual rehabilitation, physiotherapy

## Abstract

The acute phase of COVID-19 often leaves patients with persistent pulmonary deficits. Pulmonary Rehabilitation (PR) has been recommended as an essential part of post-acute COVID-19 management. In light of the global pandemic, telerehabilitation has been increasingly employed to deliver PR. This systematic review aimed to evaluate the effectiveness of telemedicine-driven PR in patients recovering from the acute phase of COVID-19, assessing variations in telerehabilitation practices and identifying the degree of change in mental health, physical health, quality of life, and lung function. A systematic search was conducted across PubMed, Web of Science, Cochrane, and Scopus up until April 2023. Studies focusing on telerehabilitation in PR for post-acute COVID-19 patients with outcomes including pulmonary function, exercise capacity, and quality of life were included after careful assessment of this study’s protocol. The selection process involved careful scrutiny of abstracts and full texts, and the quality assessment was performed using the National Heart, Lung, and Blood Institute (NHLBI) tool. Seven studies, published between 2021 and 2022, involving a total of 412 patients, were included. The evaluated telerehabilitation programs stretched between 4 and 10 weeks, involving a mobile app or video connection with the patient, integrating a mix of aerobic and resistance training, breathing exercises, functional activities, and muscle strengthening. Findings revealed that telemedicine-driven PR significantly improved physical health, measured by the step test score (73 vs. 71), 6MWD (30.2 vs. 17.1) and BPAQ, mental health evaluated by SF-12 (6.15 vs. 4.17) and PHQ-4, quality of life measured by the SF-12 (7.81 vs. 3.84), SGRQ (31.5 vs. 16.9), and CAT scores, and some parameters of pulmonary function in post-acute COVID-19 patients (mMRC, STST, and MVV). This review substantiates the potential of telemedicine-driven PR to improve various health outcomes in post-acute COVID-19 patients. The findings underscore the importance of integrating telerehabilitation into the management of post-acute COVID-19 and call for further exploration of its long-term effects, cost-effectiveness, and best practices.

## 1. Introduction

The SARS-CoV-2 virus, responsible for the COVID-19 pandemic, has posed significant challenges to healthcare systems globally since its emergence in late 2019 [1,2]. While considerable attention has been devoted to acute care management, the long-term sequelae of the disease have gradually become a subject of concern [3,4,5]. Among these, pulmonary complications stand out due to the virus’s primary respiratory tract involvement. In particular, many patients recovering from acute COVID-19 manifest persistent respiratory symptoms and functional impairment as part of the long COVID syndrome, necessitating comprehensive care strategies, including, but not being limited to, pulmonary rehabilitation (PR) [6].

Pulmonary rehabilitation is a multidisciplinary approach to care, promoting long-term adherence to health-enhancing behaviors. It combines exercise training, education, nutrition advice, and psychosocial support, aiming to improve the physical and psychological condition of individuals with chronic respiratory disease [7]. Studies have previously demonstrated the effectiveness of PR in improving the quality of life and functional status of patients with chronic obstructive pulmonary disease (COPD) and interstitial lung disease (ILD) [8,9]. This evidence has led to the adoption of PR in the management of post-acute COVID-19 patients, even though its implementation during a pandemic presents unique challenges.

Amidst the pandemic, healthcare systems have witnessed a rapid and significant shift toward telemedicine, primarily driven by the need for social distancing to limit disease transmission [10]. Telemedicine offers a platform for remote patient monitoring, consultations, and interventions, thereby reducing unnecessary hospital visits and potentially limiting healthcare-associated infection risks. It has been utilized in various fields of medicine, including chronic disease management and post-acute care, with evidence suggesting its efficacy and cost-effectiveness [11,12].

In the field of PR, the transition to telemedicine-delivered programs (telerehabilitation) is gaining momentum, particularly due to pandemic-related restrictions. Telerehabilitation can be an alternative rehabilitation approach that, using digital communication technology, allows both assessment and remote monitoring of patients during physical therapy efficiently and safely [13]. Studies have indicated that telerehabilitation may be as effective as traditional face-to-face inpatient PR, providing similar improvements in exercise capacity and quality of life in patients with COPD [14,15]. However, the application and effectiveness of telerehabilitation in patients recovering from acute COVID-19 have not been thoroughly investigated. Given the ongoing need and increasing use of telerehabilitation in PR for COVID-19 survivors, it is crucial to synthesize the existing evidence to guide clinical practice and future research. Therefore, the current study is designed as a systematic review of telemedicine-driven PR in patients recovering from acute COVID-19.

The primary hypothesis of the current study is that telerehabilitation can offer effective PR to post-acute COVID-19 patients, leading to improvements in pulmonary function, exercise capacity, and quality of life. This study’s objectives are to systematically identify, review, and analyze the existing literature on the subject. It aimed to evaluate the effects of telerehabilitation on clinical outcomes, understand the variations in telerehabilitation practices, and elucidate the potential barriers and facilitators to its implementation in this patient population. The results are expected to contribute to an informed approach to managing patients recovering from acute COVID-19 and provide guidance on the utilization and improvement of telemedicine in PR.

## 2. Materials and Methods

### 2.1. Protocol and Registration

This systematic review was executed in March 2023 by probing four electronic databases: PubMed, Web of Science, Cochrane, and Scopus. The current study included the literature published up until April 2023. The search strategy employed medical subject headings (MeSH) keywords, such as “COVID-19”, “SARS-CoV-2”, “telemedicine”, “telerehabilitation”, “pulmonary rehabilitation”, “post-acute COVID-19”, “exercise capacity”, “virtual rehabilitation”, “pulmonary function”, and “quality of life”. The search was limited to English-language journal articles.

This review adhered to the Preferred Reporting Items for Systematic Reviews and Meta-Analyses (PRISMA) guidelines [16] and the International Prospective Register of Systematic Reviews (PROSPERO) criteria [17]. A structured and systematic search strategy was implemented to identify relevant scientific papers examining the use and effectiveness of telerehabilitation in post-acute COVID-19 patients. The systematic review was registered on the Open Science Framework (OSF) platform [18], with the registration code (OSF.IO/3J8WB).

The current systematic review aimed to explore and address several research questions that assess the effectiveness and barriers of telerehabilitation in PR for post-acute COVID-19 patients. The main research question aimed to determine the effects of telerehabilitation on pulmonary function, exercise capacity, and quality of life in these patients. It was also set to examine variations in telerehabilitation practices and potential barriers and facilitators to its implementation.

### 2.2. Eligibility Criteria

The selection process began with the removal of duplicate entries, followed by a careful evaluation by two independent researchers of each abstract to assess its relevance to the research questions. Subsequently, a comprehensive review of the entire text was conducted for the remaining articles to ensure that they met the inclusion criteria. Additionally, an in-depth analysis of the reference lists of the selected papers was performed by two independent researchers, aiming to identify any pertinent literature that may have been overlooked during the initial search. Regarding the comparisons considered in this study, telerehabilitation was compared with face-to-face treatments, as well as telerehabilitation versus no treatment or basic care.

The inclusion criteria for studies in the systematic review were as follows: (1) studies addressing telerehabilitation in PR for post-acute COVID-19 patients; (2) clinical outcome measures including but not limited to pulmonary function, exercise capacity, and quality of life; (3) detailed description of the telerehabilitation program. Conversely, the exclusion criteria were as follows: (1) studies not addressing PR in post-acute COVID-19 patients; (2) studies lacking relevant data on clinical outcomes; (3) articles where telerehabilitation was not explicitly described; (4) studies involving other rehabilitation programs designed for post-COVID-19; (5) studies that were carried during the acute phase of COVID-19; (6) case reports, proceedings, reviews, commentaries, and letters to the editor were also excluded.

### 2.3. Data Collection Process

The initial search yielded a significant number of studies, of which a set number were identified as duplicates. After excluding non-relevant papers based on their abstracts, two authors scrutinized the remaining full-text articles for relevance, while a third author performed the triple check. Using the Study Quality Assessment Tools from the National Heart, Lung, and Blood Institute (NHLBI) [19], two investigators separately appraised the studies and recorded their conclusions. Data considered for extraction comprised the following: study and author information; country of publication; year of publication; study design; quality of the study; number of patients; average age of patients; gender; COVID-19 severity; length of hospitalization; program exercises; program duration; program schedule; rehabilitation protocol features; quality of life domains; and pulmonary function.

The Quality Assessment Tool for Observational Cohort and Cross-Sectional Studies was employed to evaluate the included articles [20]. Each question within the tool received a score of 1 for “Yes” responses and 0 for “No” and “Other” responses to determine the final performance score. Research with scores from 0 to 4 was labeled as poor quality, those scoring between 5 and 9 as fair quality, and those with a score of 10 or above were deemed excellent quality. To minimize bias and enhance reliability, two researchers independently assessed the quality of the selected articles.

### 2.4. Risk of Bias

Publication bias was examined by creating a funnel plot, where the standard error of the log odds ratio was plotted against its corresponding log odds ratio. The symmetry of the plot was visually examined and further assessed using Egger’s regression test, with a *p*-value of <0.05, indicating significant publication bias. A sensitivity analysis was also conducted by removing one study at a time and recalculating the pooled odds ratios to evaluate the robustness of the results on the pulmonary function and to examine the impact of individual studies on the overall effect size.

## 3. Results

### 3.1. Study Characteristics

**Figure 1:**
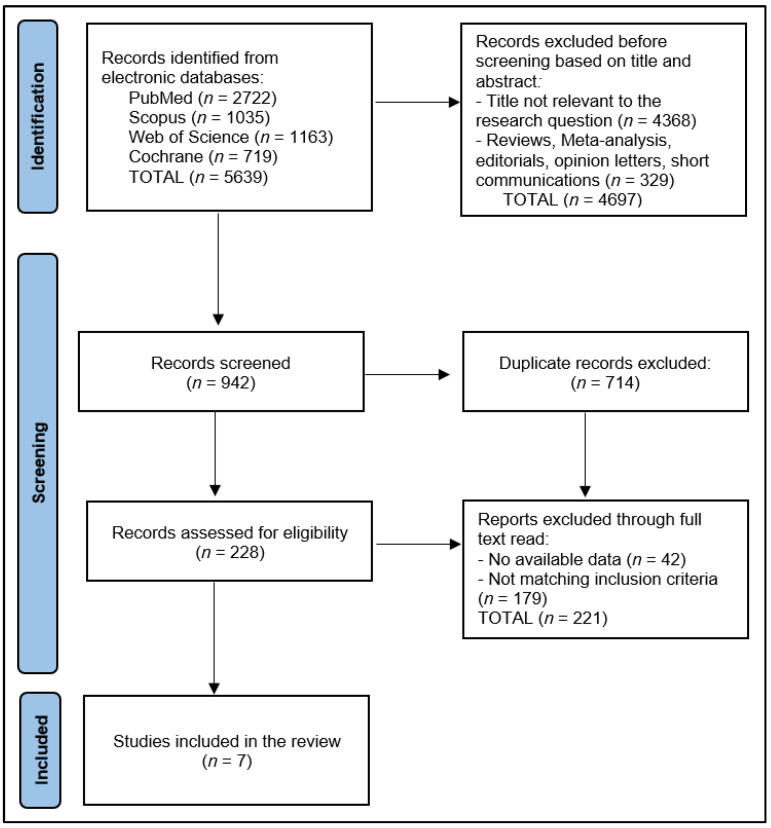
PRISMA Flow Diagram.

The systematic review assessed seven studies, as described in Figure 1, that were conducted in various countries spanning Chile, Belgium, Turkey, USA, China, and Canada [21,22,23,24,25,26,27], as presented in Table 1. This geographical distribution indicates a worldwide engagement in this research area. All studies were published within the timeframe from 2021 to 2022, indicating a recent interest in exploring the effectiveness of telemedicine-driven pulmonary rehabilitation in the post-acute phase of COVID-19. Two distinct study designs were employed across the studies, with prospective cohort studies and randomized trials, each being utilized by about half of the studies included in this review.

Assessment of study quality revealed that the majority of the studies, four in number, were categorized as ‘Good’ quality [21,22,24,27]. These were the studies led by Dalbosco-Salas [21], Martin [22], Hameed [24], and Tanguay [27]. The remaining three studies, conducted by Pehilvan [23], Li [25], and Capin [26], were categorized as ‘Excellent’. Interestingly, all of the ‘Excellent’ quality studies were randomized trials conducted in the latter year of the timeframe, in 2022 [23,25,26].

**Figure 2:**
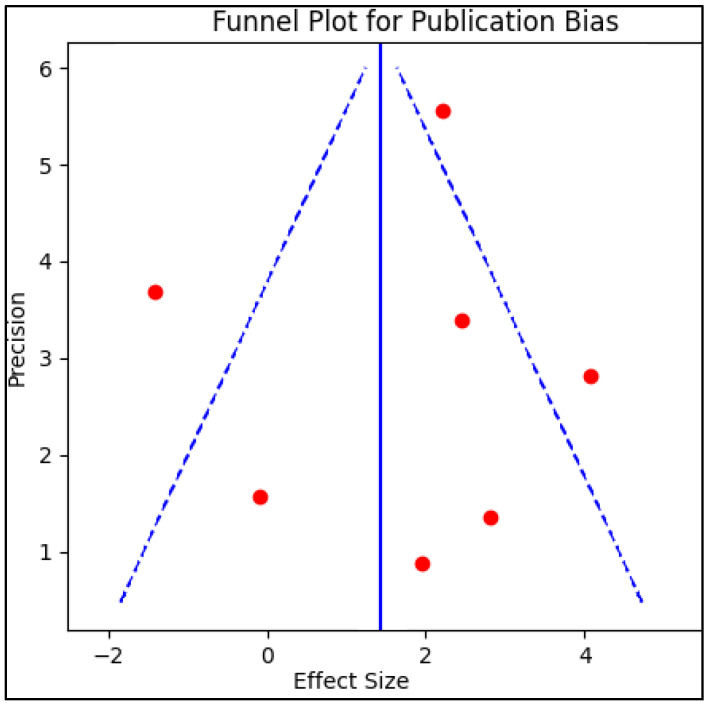
Funnel plot for publication bias.

The systematic review incorporated seven studies, enlisting a total of 412 patients who underwent telemedicine-driven pulmonary rehabilitation after the acute phase of COVID-19 [21,22,23,24,25,26,27], with good reliability for publication bias, as presented in Figure 2. The cohort sizes varied substantially across the studies, with the smallest involving 7 patients [27] and the largest encompassing 119 participants [25]. The average age of the participants differed across the studies, ranging from the lowest mean age of 42 years reported by Tanguay et al. [27] to the highest mean of 62 years reported in the control group of Martin et al.’s study [22]. The age differences within each study were also variable, suggesting potentially different age-related effects on the outcome of rehabilitation.

The gender distribution across all studies displayed a higher percentage of males. However, the proportion varied, with the lowest proportion of male patients observed in Hameed et al.’s study at 36% [24] and the highest in the telemedicine group of Pehilvan et al.’s study, which reported 82.0% male patients [23]. The severity of COVID-19 among the patients was not uniform across the studies. Two studies, Dalbosco-Salas et al.’s and Tanguay et al.’s, reported no severe COVID-19 cases [21,27]. In contrast, the highest proportion of severe cases was reported by Martin et al. at 48% [22]. The remaining studies reported varying severity levels, ranging from 9% to 32% [23,24,25,26]. Regarding the length of hospitalization, the data were not consistently reported across all studies. Where reported, there were differences between control and intervention groups and across studies. For instance, Capin et al. reported the shortest hospitalization durations at eight days for the virtual group and five days for the control group [26]. Conversely, Dalbosco-Salas et al. reported a longer average length of hospitalization at 29.9 days [21], as described in Table 2.

### 3.2. Rehabilitation Programs

A diverse range of rehabilitation programs for post-acute COVID-19 patients were evaluated, as described in Table 3. These programs integrated a mix of aerobic and resistance training, breathing exercises, functional activities, and other techniques tailored to each study’s patient population and study design. The duration of the programs varied between 4 weeks [24] and 10 weeks [26], suggesting that the length of telemedicine-driven pulmonary rehabilitation could be adjusted based on patients’ needs and recovery speed. The schedule of the programs was also flexible, with some programs offering sessions three times per week [21,23,26], while others adjusted the frequency as the weeks progressed [26] or offered daily sessions [27]. The length of each session also varied, ranging from 30 min [27] to up to 60 min [24,25].

The rehabilitation protocols were designed to address the unique needs of COVID-19 patients, and they often incorporated moderate- to high-intensity training [21,26], as well as resistance training using elastic bands [21] or at-home materials [22]. Breathing exercises were a consistent feature across multiple studies, designed to improve lung function [21,23,25,26]. Other studies incorporated such techniques as paced walking or running, range of motion exercises, and standing squats [23]. Hameed et al.’s protocol was unique in its phased approach, incrementing the number of workout cycles each day and progressing to community-level exercise in the final phase [24]. Some programs also included components to promote patient engagement and adherence, such as lifestyle counseling, motivational interviews, and weekly phone calls [26].

### 3.3. Rehabilitation Measures and Outcomes

The results demonstrated the effectiveness of telemedicine-driven pulmonary rehabilitation in enhancing various health outcomes in post-acute COVID-19 patients, as presented in Table 4. Measures of physical health, mental health, quality of life, and pulmonary function were assessed across the included studies. In terms of physical health, measures, such as the VAS fatigue score, VAS pain score, and six-minute walking distance (6MWD), demonstrated significant improvements in some studies [21,23,25]. On the contrary, Dalbosco-Salas et al. found a significant difference in VAS fatigue scores between the non-hospitalized patients (telemedicine group) and the hospitalized group, indicating a higher pain score after telerehabilitation compared with the hospitalized patients (*p* < 0.001) [21]. However, Li et al. reported substantial improvement in 6MWD in the telerehabilitation group compared to controls (*p* < 0.001) [25]. Mental health outcomes were also significantly improved in some studies following telerehabilitation. For example, SF-36 mental domain scores in the hospitalized group significantly improved in Dalbosco-Salas et al.’s study (*p* < 0.001) [21], and there was a marked enhancement in the SF-12 mental domain scores in Li et al.’s study (*p* < 0.001) [25].

Quality of life, as measured by the SF-36 total score and SF-12 total score, showed significant improvements in the hospitalized group in Dalbosco-Salas et al.’s study (*p* < 0.001) [21] and the telerehabilitation group in Li et al.’s study (*p* < 0.001) [25]. Pulmonary function, as assessed by such measures as the mMRC dyspnea score, VAS dyspnea score, and STST change, showed varied results across studies. For example, Dalbosco-Salas et al. reported significant improvements in mMRC dyspnea scores and STST between the non-hospitalized and hospitalized groups (*p* < 0.001) [21]. In contrast, Martin et al.’s study found no significant difference in VAS dyspnea scores between the telerehabilitation and control groups (*p* = 0.966) but did observe a significant difference in STST change (*p* = 0.004) [22].

## 4. Discussion

### 4.1. Summary of Evidence

This systematic review reveals a growing interest in telemedicine-driven pulmonary rehabilitation following the acute phase of COVID-19, although, as the pandemic reaches an end, it will be interesting to observe this trend in the upcoming years. The studies included in this review were conducted across diverse geographical settings, further supporting the potential global applicability of telemedicine interventions in post-acute COVID-19 management. It seems that regardless of the study design, the integration of telerehabilitation in post-acute care significantly improved several health outcomes among COVID-19 patients. These improvements were observed in physical and mental health, quality of life, and pulmonary function, albeit with variations across different studies.

In terms of rehabilitation protocols, a significant amount of heterogeneity was observed among the studies. This could be reflective of the individualized approach necessary for addressing the distinct needs of post-acute COVID-19 patients, as well as the absence of a unified protocol for telerehabilitation in this setting. The protocols were designed to incorporate a balance of aerobic and resistance training, breathing exercises, and functional activities, often accompanied by counseling or motivational measures to promote adherence. The intensity, duration, and frequency of the sessions varied widely, suggesting the need for further studies to identify optimal parameters for telerehabilitation programs in this population.

Different rehabilitation protocols emphasize various exercises, including deep breathing, inspiratory muscle training, and breathing control techniques, although the majority were performed in a hospital or institutional setting, contrary to our study [28,29,30,31]. These protocols often also incorporate physical exercises for strength and endurance, which help in functional improvement and disability reduction [32,33]. However, the question that arises is what degree of change and disability reduction is dependent on the PR setting. A holistic approach, encouraged by the American Thoracic Society (ATS) and the European Respiratory Society (ERS), expands beyond mere physical exercises, embracing comprehensive patient evaluation and lifestyle modifications [34]. Some studies have combined their rehabilitation protocols with educational sessions to address such issues as dyspnea, cough, fatigue, anxiety, memory, and daily activity management [35,36]. These integrative approaches have demonstrated significant improvements in functional abilities, patient’s quality of life, and reintroduction into professional life.

Around 90% of COVID-19 patients in hospitals deal with debilitating lung effects, indicating the importance of physical and respiratory rehabilitation [37,38]. The most common symptoms include dyspnea, fatigue, and exercise intolerance. Thus, telerehabilitation, offering physiotherapy remotely, is a suitable option to address these issues, particularly during social distancing, while its convenience also encourages patient adherence [39]. Improvements in physical health outcomes, such as fatigue, pain, and exercise capacity, suggest that telerehabilitation can be an effective modality to enhance recovery and function after acute COVID-19. The improvements in mental health outcomes further support the potential of telerehabilitation in addressing the psychological impact of the disease, an aspect that is often overlooked in physical rehabilitation programs. The positive effect on quality-of-life measures is particularly encouraging, given the significant impact COVID-19 can have on overall well-being.

The pandemic added challenges for research and trial participation due to strict control measures and economic struggles. Randomized trials showcased a variety of participant details, disease stage, telerehabilitation methods, and the varying telemonitoring options that could have impacted outcomes, such as smartphones, video conferences, and messaging applications [40,41]. The rush to introduce remote interventions often came with inadequate implementation guidance and professional training, which was evident in some of our included studies and other trials for acute COVID-19 [25,42]. Despite the variability in the measures used to assess pulmonary function, some significant improvements were reported. It is important to highlight that the effects on pulmonary function seem to depend on the severity of the disease, with the less severe cases showing more significant improvements. Thus, one hypothesis that this study suggests is that telerehabilitation might be more effective when initiated in the early post-acute phase before severe pulmonary sequelae develop.

Moreover, further research is needed to examine the aspects of physical abilities and lung function that better represent changes during PR post-COVID-19. Notably, these parameters encompass Forced Vital Capacity (FVC), Forced Expiratory Volume in 1 s (FEV1), and Diffusing Capacity for Carbon Monoxide (DLCO), as used in other PR programs for COPD and ILD [43,44]. Furthermore, not only do these traditional lung function tests show improvement following a physical rehabilitation program, but the severity of dyspnea, or shortness of breath, also significantly decreased among adult survivors of COVID-19. This provides another practical measurement of improved respiratory function, as reducing the feeling of breathlessness is a critical component of the recovery process. In addition to the aforementioned tests, it may also be beneficial to consider other tests, such as the Total Lung Capacity (TLC) and the Peak Expiratory Flow (PEF), that may provide a more nuanced understanding of the impact of physical rehabilitation programs on lung function among COVID-19 survivors.

The quality of the studies included in this review was generally good or excellent, suggesting reliable findings. However, it is worth noting that the higher-quality studies were all randomized trials conducted in 2022, indicating that the quality of research in this area is improving. Additionally, the higher-quality studies tended to have larger sample sizes, suggesting that they may provide more reliable evidence for the effectiveness of telerehabilitation in this setting.

### 4.2. Limitations

While the findings of this review are encouraging, it is important to acknowledge its limitations. The diversity of the rehabilitation programs, their duration, intensity, and the measures used to assess outcomes across studies make it challenging to draw definitive conclusions. There was also a wide age range among the participants, which might influence the outcomes of the rehabilitation programs. The gender distribution was not balanced across studies, and the severity of COVID-19 varied, factors that might also affect the response to rehabilitation. Furthermore, many studies did not provide detailed data about the length of hospitalization, making it difficult to explore its potential impact on outcomes. Moreover, the number of included studies was relatively small, indicating a need for more high-quality research in this field.

By limiting the search to studies published in English, there may have been inadvertently introduced language bias, potentially excluding relevant studies published in other languages. In addition, this review did not include the gray literature, such as conference papers or technical reports, possibly leading to publication bias. Despite the robust measures used to assess this, the possibility of missing some relevant information cannot be entirely negated. Finally, our quality assessment was based on the National Heart, Lung, and Blood Institute (NHLBI) tool. Despite its wide acceptance and use, it possesses inherent limitations and subjectivity, which could potentially influence the conclusions drawn from this review. Future studies should strive for uniformity in their protocols and measures to allow for more direct comparisons and, potentially, meta-analyses.

## 5. Conclusions

The findings from this systematic review suggest that telemedicine-driven pulmonary rehabilitation can have beneficial effects on physical and mental health, quality of life, and pulmonary function in patients recovering from the acute phase of COVID-19. Therefore, this can be a very useful tool even beyond the pandemic, which, at the present time, is officially over. The varied interventions, ranging from aerobic and resistance training to breathing exercises, appear to contribute to improved patient outcomes. However, the significant heterogeneity in program features, duration, and intensity across the studies indicates a need for standardized telerehabilitation protocols in this patient population. Furthermore, these results should be interpreted with caution due to differences in study populations and varying severity of COVID-19 among participants. Future high-quality, randomized controlled trials are warranted to further examine the effectiveness and optimal delivery methods of telemedicine-driven pulmonary rehabilitation in post-acute COVID-19 patients, as well as in other pulmonary diseases.

## Figures and Tables

**Table 1:** Study characteristics.

Study and Author	Country	Study Year	Study Design	Study Quality
1 Dalbosco-Salas et al. [21]	Chile	2021	Prospective Cohort	Good
2 Martin et al. [22]	Belgium	2021	Prospective Cohort	Good
3 Pehilvan et al. [23]	Turkey	2022	Randomized Trial	Good
4 Hameed et al. [24]	USA	2021	Prospective Cohort	Good
5 Li et al. [25]	China	2022	Randomized Trial	Excellent
6 Capin et al. [26]	USA	2022	Randomized Trial	Excellent
7 Tanguay et al. [27]	Canada	2021	Prospective Cohort	Good

**Table 2:** Characteristics of patients in the included studies.

Study Number	Number of Patients	Average Age (Years)	Gender (Men, %)	COVID-19 Severity	Length of Hospitalization
1 Dalbosco-Salas et al. [21]	115 (58 non-hospitalized vs. 57 hospitalized)	52 vs. 59	43% (24% vs. 61%)	Severe—0.0%	29.9 days
2 Martin et al. [22]	27 (14 telerehabilitation vs. 13 controls)	61 vs. 62	62% (79% vs. 46%)	Severe—48.1%	16.3 days vs. 16.1 days
3 Pehilvan et al. [23]	34 (17 telerehabilitation vs. 17 controls)	51 vs. 43	74% (82% vs. 65%)	Severe—8.8%	NR
4 Hameed et al. [24]	69 (44 virtual rehabilitation vs. 25 in-person rehabilitation therapy)	60 vs. 57	36% (57% vs. 24%)	Severe—31.8%	14 days vs. 39 days
5 Li et al. [25]	119 (59 telerehabilitation vs. 60 controls)	50 vs. 52	45% (46% vs. 43%)	Severe—31.9%	28.6 days vs. 23.7 days
6 Capin et al. [26]	41 (28 virtual vs. 13 controls)	52 vs. 54	54% (54% vs. 62%)	Severe—21.9%	8 days vs. 5 days
7 Tanguay et al. [27]	7 (before vs. after rehabilitation)	42	47%	Severe—0.0%	NR

NR—Not Reported.

**Table 3:** Characteristics of rehabilitation programs.

Study Number	Program Exercises	Program Duration	Program Schedule	Rehabilitation Protocol Features
1 Dalbosco-Salas et al. [21]	Aerobic, resistance training, and breathing exercise	9 weeks	3 sessions/week; duration: 40 min	Moderate- to high-intensity training. Resistance training was performed with elastic bands.
2 Martin et al. [22]	Resistance training, muscle training	6 weeks	2 sessions/week; duration: 50 min	Fixed intensity of the endurance training on a 6-point intensity score. Muscle training was performed with materials available at home (2–3 series of 8–12 repetitions for each exercise).
3 Pehilvan et al. [23]	Paced running/walking in the corridor, breathing exercises, active cycle of breathing technique, range of motion exercise, and standing squats.	6 weeks	3 sessions/week	10 repetitions of each exercise or modified according to the level of fatigue.
4 Hameed et al. [24]	Sit to stand, marching in place, shoulder scaption, standing heel raises, side stepping, wall pushups.	4 weeks	4 phases; duration: 30–60 min	Phase one required one workout cycle, four times a day, for seven days. Phase two required two workouts each day with a rest day on the fourth of seven days. Phase three required three workout cycles, twice daily for three days, followed by a day of rest and another three days. Phase four recommended community-level exercise.
5 Li et al. [25]	Aerobic, resistance training, and breathing exercise.	6 weeks	4 sessions/week; duration: 40–60 min	Breathing control and thoracic expansion, aerobic exercises, lower-limb exercises (resistance training)—all exercises scheduled to increase in intensity.
6 Capin et al. [26]	Aerobic, high-intensity training, breathing and clearance techniques, balance exercises, and functional activities.	10 weeks	12 sessions: 3/week in the first week, 2/week in weeks 2–4, 1/week in weeks 5–6, and 1 recall visit during weeks 9–10	High-intensity strength training included breathing and compensating strategies. Balance, functional, and aerobic exercises were performed. Stretching ensured flexibility, while lifestyle counseling and motivational interviews kept participants engaged. Health in Motion enabled self-directed intervention outside of supervised sessions. Educational handouts were used to teach exercise, and weekly phone calls monitored progress and program adherence.
7 Tanguay et al. [27]	Aerobic and resistance training	8 weeks	7 sessions/week; duration: 30 min	Aerobic training consisted of 15 min of intense cycling, steps, or walking. Resistance training followed, with 1–2 sets of 8–10 repetitions at 30%–80% of the repetition maximum. Balance training—walking with obstacles, changing direction, or stepping on unsteady surfaces—improved stability and coordination.

**Table 4:** Rehabilitation measures and outcomes (after intervention).

Study Number	Physical Health	Mental Health	QOL	Pulmonary Function
1 Dalbosco-Salas et al. [21]	VAS fatigue score: 3 (0–5) non-hospitalized vs. 1 (0–3) hospitalized (*p* < 0.001)	SF-36 mental domain: 51.0 non-hospitalized vs. 63.7 hospitalized (*p* < 0.001)	SF-36 total score: 39.6 non-hospitalized vs. 58.9 hospitalized (*p* < 0.001)	mMRC dyspnea score: 2 (1–3) non-hospitalized vs. 1 (0–2) hospitalized (*p* < 0.001); STST: 18.9 vs. 29.2 (*p* < 0.001)
2 Martin et al. [22]	NR	NR	NR	VAS dyspnea score: 5 (3–8) TR vs. 5 (1–10) controls (*p* = 0.966); STST change: 10 (5–19) TR vs. 5 (−4–11) controls (*p* = 0.004)
3 Pehilvan et al. [23]	VAS pain score: 2.47 (0–8) TR vs. 1.76 (0–7) controls (*p* = 0.039) VAS fatigue score: 1.29 (0–3) TR vs. 1.47 (0–5) controls (*p* = 0.782)	BDI score: 3.2 TR vs. 4.1 controls (*p* = 0.623)	SGRQ total score: 31.5 TR vs. 16.9 controls (*p* = 0.033)	mMRC dyspnea score: 1 (0–2) TR vs. 0 (0–2) controls (*p* = 0.066)
4 Hameed et al. [24]	Step test score: 73 ± 26 virtual vs. 71 ± 30 in-person (*p* < 0.001)	PHQ-4: 1 (4) virtual vs. 0 (1) in-person (*p* = 0.020)	NR	STST: 13 ± 3 virtual vs. 12 ± 1 in-person (*p* < 0.001)
5 Li et al. [25]	6MWD: 80.2 TR vs. 17.1 controls (*p* < 0.001)	SF-12 mental domain: 6.15 TR vs. 4.17 controls (*p* < 0.001)	SF-12 total score: 7.81 TR vs. 3.84 controls (*p* < 0.001)	MVV: 14.5 TR vs. 5.6 controls (*p* = 0.005); mMRC dyspnea: 90.4 TR vs. 61.7 controls (*p* < 0.001)
6 Capin et al. [26]	30 seconds chair stand: 11 (3) TR vs. 12 (3) controls (*p* = 0.730); TUG: 9 (3) TR vs. 10 (3) controls (*p* = 0.790)	3-item LS: 4 (1) TR vs. 5 (2) controls (*p* = 0.510)	PHQ8 score: 6 (4) TR vs. 8 (6) controls (*p* = 0.370)	mMRC dyspnea score: 3 (1) TR vs. 3 (1) controls (*p* = 0.430)
7 Tanguay et al. [27]	EQ-VAS: improvement between 10–45 points BPAQ: increase from low to moderate level	Anxiety/Depression: significant decrease	CAT score: increase by 30 points after 8 weeks	Borg dyspnea scale: average decrease by 4 points (from 7 to 3)

NR—Not Reported; QOL—Quality of life; SF—Short form; VAS—Visual analog scale; mMRC—Modified medical research council; STST—Sit-to-stand test; BDI—Beck depression inventory; SGRQ—Saint George respiratory questionnaire; PHQ-4—Patient health Questionnaire 4; TR—Telerehabilitation; 6MWD—Six min walking distance; MVV—Maximum voluntary ventilation; PHQ8—Patient health questionnaire 8; CAT—COPD assessment test; BPAQ—Baecke physical activity questionnaire.

## Data Availability

Not applicable.
